# Interventional Pneumology in Pulmonary Bleeding; A Review: From the Bronchus to the Vessel

**DOI:** 10.1155/DTE.4.19

**Published:** 1997

**Authors:** Christian Witt, Paul Romaniuk, Bernd Schmidt, Anika Geisler, Katrin Klein, Ingo Fietze, Adrian Constantin Borges, Uta Liebers, Wolf Dörffel, Gert Baumann

**Affiliations:** Div. of Pneumology Dept. of Internal Medicine I Div. of Interventional Radiology of Medical School (Charité) Humboldt-University of Berlin, Schumannstr. 20/21 Berlin 10117 Germany

## Abstract

Interventional pneumology includes both bronchological and vascular methods of diagnosis and therapy, especially in emergency situations such as pulmonary hemorrhage. In massive pulmonary hemorrhage bronchological diagnosis is required to determine the site and extent of bleeding, as well as angiography of bronchial arteries, and of pulmonary arteries. Bronchus occlusion by aid of balloon catheter or double lumen tube are holding measures until definitive surgery or embolization of bronchial or pulmonary arteries can be performed. The paper suggests a close relationship between bronchoscopic and angiographic diagnosis and therapy in case of severe pulmonary bleeding.


*Diagnostic and Therapeutic Endoscopy*, Vol. 4, pp. 19–28
Reprints available directly from the publisher
Photocopying permitted by license only

© 1997 OPA (Overseas Publishers Association)
Amsterdam B.V. Published in The Netherlands
by Harwood Academic Publishers
Printed in Singapore

### Interventional Pneumology in Pulmonary Bleeding A Review: From the Bronchus to the Vessel

CHRISTIAN WITT *, PAUL ROMANIUK, BERND SCHMIDT, ANIKA GEISLER, KATRIN KLEIN, INGO FIETZE, ADRIAN CONSTANTIN BORGES, UTA LIEBERS, WOLF DÖRFFEL and GERT BAUMANN

*Div. of Pneumology, Dept. of Internal Medicine I, Div. of Interventional Radiology of Medical School (Charité), Humboldt-University of Berlin, Schumannstr. 20/21, 10117 Berlin, Germany*

*(Received 27 August 1996; In final form 5 December 1996)*

**Interventional pneumology includes both bronchological and vascular methods of diagnosis and therapy, especially in emergency situations such as pulmonary hemorrhage. In massive pulmonary hemorrhage bronchological diagnosis is required to determine the site and extent of bleeding, as well as angiography of bronchial arteries, and of pulmonary arteries. Bronchus occlusion by aid of balloon catheter or double lumen tube are holding measures until definitive surgery or embolization of bronchial or pulmonary arteries can be performed. The paper suggests a close relationship between bronchoscopic and angiographic diagnosis and therapy in case of severe pulmonary bleeding.**

*Keywords:* Hemoptysis, Diagnosis, Bronchoscopy, Therapy, Bronchial artery embolization

### PULMONARY HEMORRHAGE – INTRODUCTION

The etiological spectrum of hemoptysis has been changing over the last few decades. Tuberculosis was the most frequent cause of pulmonary hemorrhage 40 years ago. Today inflammatory and neoplastic diseases have taken over this position, because of the decrease of tuberculosis. The incidence of pulmonary hemorrhage in the case of vascular abnormality (e.g. arteriovenous fistulae, collateral pulmonary and bronchial arteries in patients with cyanotic heart disease) and cardiac diseases is about 9% (Johnston and Reisz, 1989). Pulmonary bleeding is classified in a three-step score (I $<$ 20 ml/d, II 20–200 ml/d, III $>$ 200 ml/d). In tuberculosis and bronchiectasis approximately 40% of all pulmonary bleeding episodes are massive (degree III). In bronchitis and bronchial carcinoma massive bleeding (III) is found in 13%, or 5 to 7% respectively (Conlan *et al.*, 1983). Hemoptysis with loss of blood $>$ 600 ml is associated with a mortality of up to 50% (Corey and Hla, 1987).

The sources of bleeding are often bronchial or intercostal arteries, and rarely pulmonary arteries.

* Corresponding author. Tel.: +49 (030) 2802 4012. Fax: +49 (030) 2802 8991.

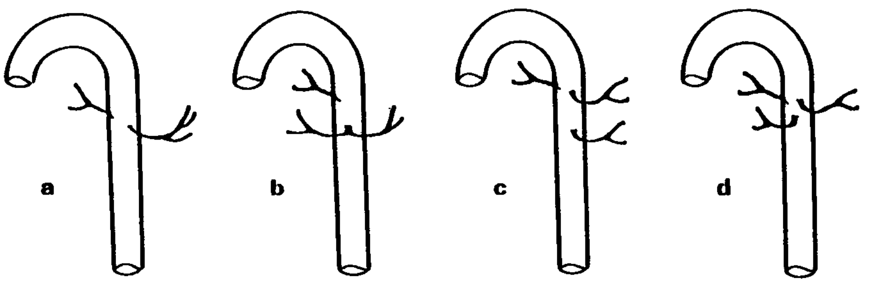

FIGURE 1 Most common origins of bronchial arteries, based on angiographic findings in 72 patients (a: 31%, b: 25%, c: 13%, d: 12%). Reprinted from Uflacker.

Bronchial arteries accompany the bronchi to the level of the respiratory bronchioles, where they anastomose with pulmonary vessels. The origins of bronchial arteries show wide variation, which can, in 80% of the cases, be described by four patterns (Fig. 1). In 40% two bronchial arteries come from the left side and one intercosto-bronchial trunc from the right side of the aorta at the fifth or sixth dorsal vertebra ($D_5$, $D_6$). In general, many of the right bronchial arteries originate together with intercostal arteries (intercosto-bronchial trunc) from the posterio-lateral side of the aorta, while left bronchial arteries are more likely to originate directly from the anterior aortic wall. Abberant origins (20%) are subclavian, internal mammary, pericardiophrenic, inferior phrenic, and thyreocervical arteries or abdominal aorta. This anatomic variation may complicate probing of bronchial arteries (Cauldwell *et al.*, 1948; Deffabach *et al.*, 1987; Uflacker *et al.*, 1985).

### DIAGNOSIS OF PULMONARY HEMORRHAGE

Diagnosis of pulmonary hemorrhage always starts with taking a history (quantity, colour, aspect, duration and time of the bleeding). This includes information about possible underlying diseases (inflammation, tumor, cardio-pulmonary malformations, bronchiectases, heart failure, rheumatologic diseases like Wegener's disease, Goodpasture syndrome, and acute lupus pneumonitis) and coagulation problems. Subsequent clinical examination comprises blood pressure, ventilation, blood gases, and the current status, e.g. colour of the expectorated blood.

Localized lung processes (infiltrations, circular foci, caverns) can be found in only half of patients on chest X-ray (Poe *et al.*, 1988). High resolution computed tomography (HRCT) is helpful to detect small lesions in 50% of the patients with normal chest X-ray (McGuinness *et al.*, 1994; Naidich *et al.*, 1990). This is valid for peripheral diseases and parenchymatous lesions which are not accessible by means of diagnostic bronchoscopy (Jackson *et al.*, 1985). In any case imaging is useful to show the extent of the bleeding and resulting condition of parenchyma and airways.

### BRONCHOSCOPY AND ANGIOGRAPHY IN PULMONARY HEMORRHAGE

Diagnostic bronchoscopy provides information about localization (segment or lobe), activity, and endobronchial causes of the bleeding (Lederle *et al.*, 1989). In smokers over 40 years ($>40$ pack-years) suffering from hemoptysis for more than one week, a lung tumor is most likely to be the cause of bleeding and therefore has to be excluded (O'Neil and Lazarus, 1991). In case of massive pulmonary hemorrhage it can be extremely difficult to determine the site of bleeding, because blood can be found in every part of the lung. Under such circumstances bronchoscopy reveals the source of bleeding in only 43% (Knott-Craig *et al.*, 1993). Even if X-ray or CT scan show a suspect lesion the findings should be confirmed by bronchoscopy, because a radiographically visible lesion might not be the actual source of bleeding (Figs. 2a and 2b) (Witt *et al.*, 1995). Thoracic computed tomography and bronchoscopy are essential diagnostic procedures in pulmonary hemorrhage.

If the patient arrives with active bleeding, his clinical condition determines the course of management. In case of severe hemorrhage emergency bronchoscopy and, if necessary, bronchus occlusion are indicated. If the situation is less urgent thoracic X-ray and computed tomography should be performed prior to endoscopic intervention (Müller, 1994; Set *et al.*, 1993). If the bleeding continues bronchial-, intercostal-, or pulmonary arteriography has to be added. Origins and anatomy of bronchial arteries can be detected in retrograde aortography. Whenever a vessel is suspected to be the source of bleeding, it has to be visualized selectively. In arteriography the most important direct sign of bleeding is the extra-vasation of contrast agent (Fig. 3a). Indirect signs are hypervascularisation, atypical vessel convolutions and aneurysms (Fig. 3b) (Rabkin *et al.*, 1987; Stoll and Bettman, 1988). If a suspect vessel cannot be found at its usual anatomical place, all other known abberant origins of bronchial arteries should be checked (Newton and Preger, 1965).

### BRONCHOSCOPIC AND ANGIOGRAPHIC TREATMENT OF PULMONARY BLEEDING

Treatment of pulmonary hemorrhage depends on the intensity and cause of the bleeding. In case of intermittent low grade hemoptysis treatment of the underlying disease (tuberculosis, bronchitis,

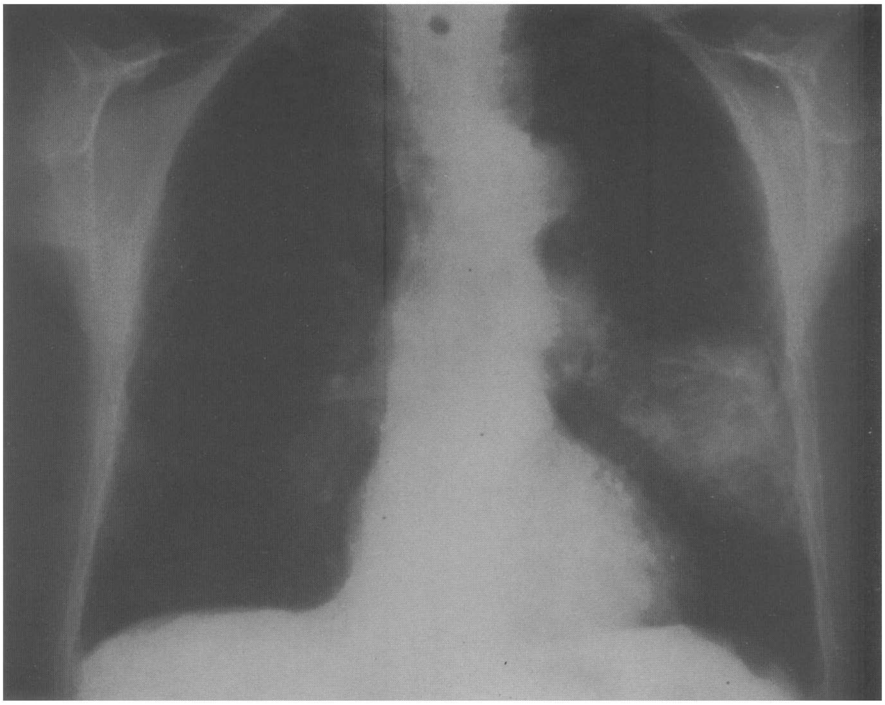

FIGURE 2a Clearly visible tumorous infiltration in projection on the left middle lung field in patient with massive pulmonary hemorrhage.

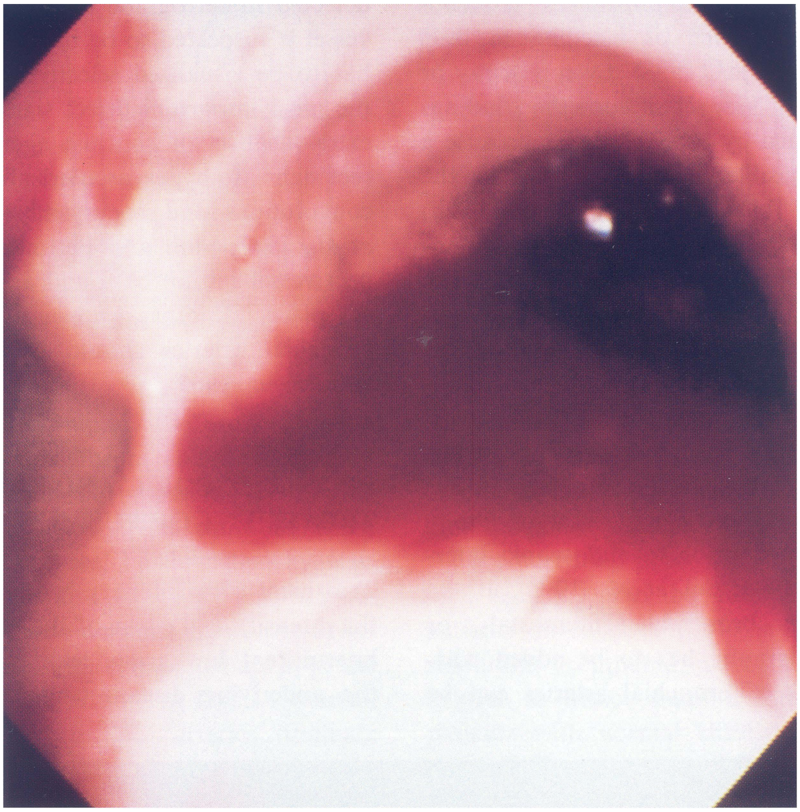

FIGURE 2b Bronchoscopy of that patient shows, that the actual source of bleeding is the right upper lobe which is without pathological findings in chest X-ray. The angiographical finding of the right bronchial artery demonstrates an angiodysplastic region in the right upper lobe, causing the massive pulmonary bleeding. This case shows the importance of bronchoscopy before both intervention (embolization) or surgical resection (Figs. 2a and 2b show findings from the same patient).

abscess, malignant tumor) may stop the bleeding. Even spontaneous resolution of the bleeding can be observed with bed rest. In massive active bleeding hemostasis has to be performed as fast as possible according to a differential therapeutic strategy. Surgical intervention is indicated if the patient is operable. Mortality after lung resection in case of bleeding tuberculosis (7.1%) is even lower than mortality after drug and interventional therapy (11.5%), and surgery is superior with regard to recurrence (Knott-Craig *et al.*, 1993).

If the patient is inoperable due to general or local reasons bronchological intervention is indicated. In rare cases the source of bleeding is visible to the endoscopist (e.g. intraluminal carcinoma). This makes topical application of vasopressor drugs and subsequent Nd : YAG-laser coagulation possible (Figs. 4a and 4b). More often the origin of bleeding will not be visible so that ballon occlusion of lobe bronchus or even main bronchus is inevitable (Fig. 5). This temporary measure should stabilize the patient's vital functions for any subsequent surgical or vascular intervention. A simple but effective tool for this purpose is the CARLENS®-tube which ensures ventilation in the contralateral bronchial system.

One of the most efficient interventions for interruption of pulmonary bleedings is artery embolization of bronchial (BAE), intercostal, or pulmonary arteries (Wholey *et al.*, 1976) (Figs. 6 and 7). Micro-metallic coils with or without

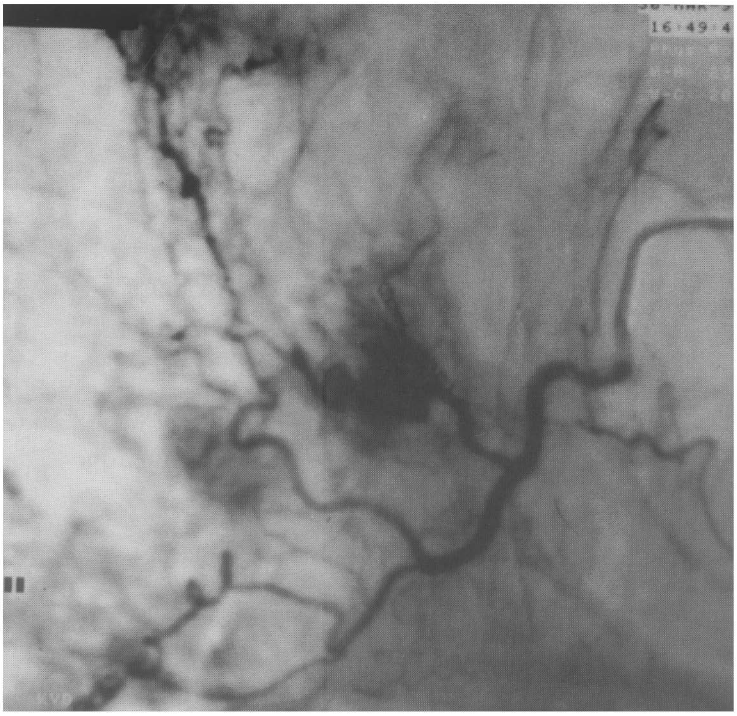

FIGURE 3a Contrast agent extravasation in recurrent bleeding (secondary findings: metal coils from previous embolization).

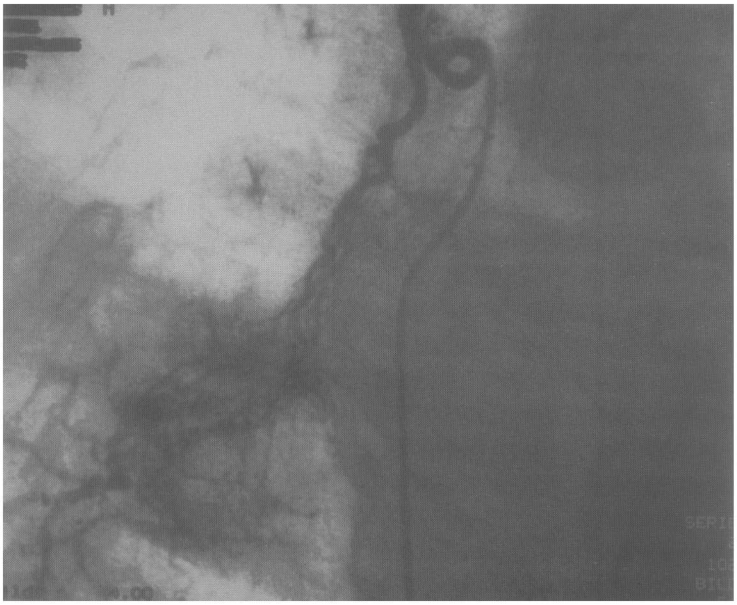

FIGURE 3b Selective demonstration of a right bronchial artery shows hypervascularisation typically in projection on the right lower lung field.

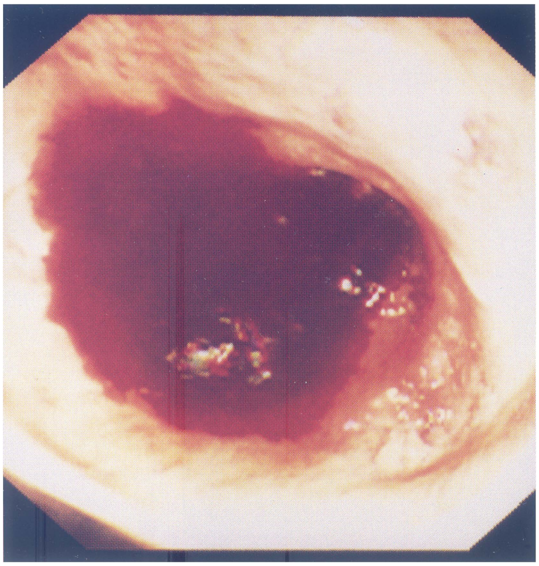

FIGURE 4a Pulmonary bleeding due to recurrence of bronchial carcinoma after pneumonectomia.

dacron fibres and gelatin sponge are most commonly used for vessel occlusions. In case of hypervascularisation the use of metallic coils should be combined with Microspheres (150 to 500 micron) or liquid occlusive agent (Ethiblock®). Mechanisms of action are mechanical occlusion, platelet aggregation or gel precipitation. Severe complications after BAE are occlusions of accessory spinal vessels with subsequent ischemia of the spinal cord (Vujic *et al.*, 1980). This may be the case in right intercostal arteries ($D_5$) or in a common right intercosto-bronchial trunc (DiChiro, 1974). Complications are encountered in 0.7% of the procedures (Lamarque and Senac, 1979). Remy *et al.* (1977) presented successful hemostasis in 84% of the patients treated in 1977. Similar results have been achieved by other groups dealing with inflammatory diseases (tuberculosis, bronchiectases, abscess, aspergilloma). Post-interventional

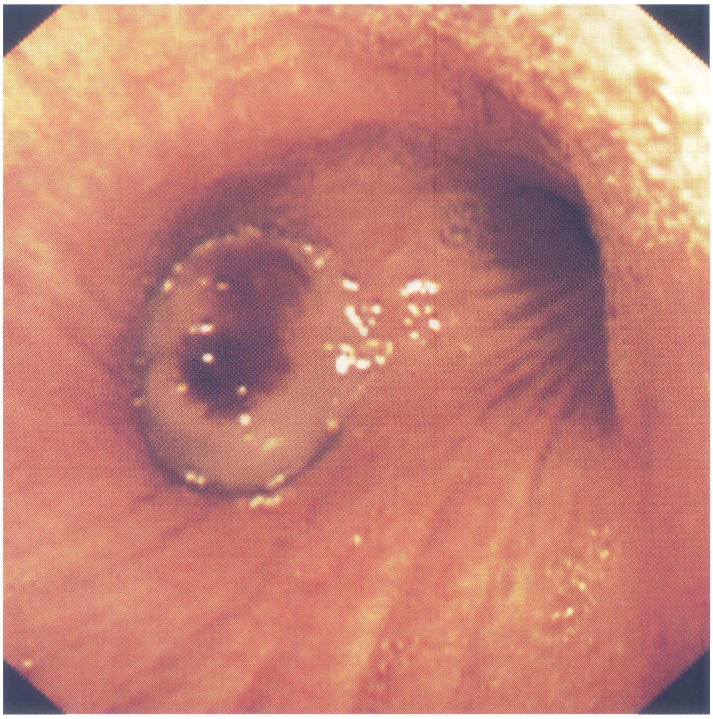

FIGURE 4b Arrest of bleeding due to Nd-YAG-laser radiation in case of visible source of bleeding.

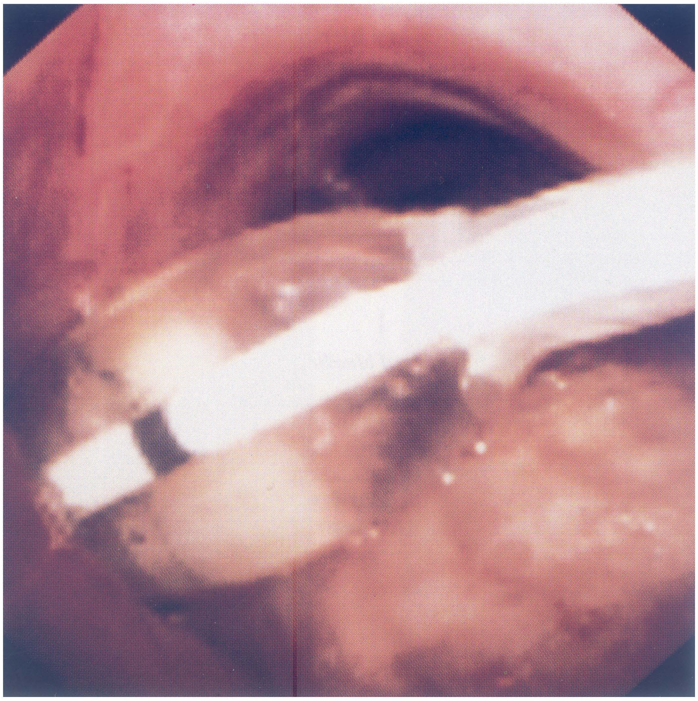

FIGURE 5 Balloon occlusion of the bronchus in case of bleeding.

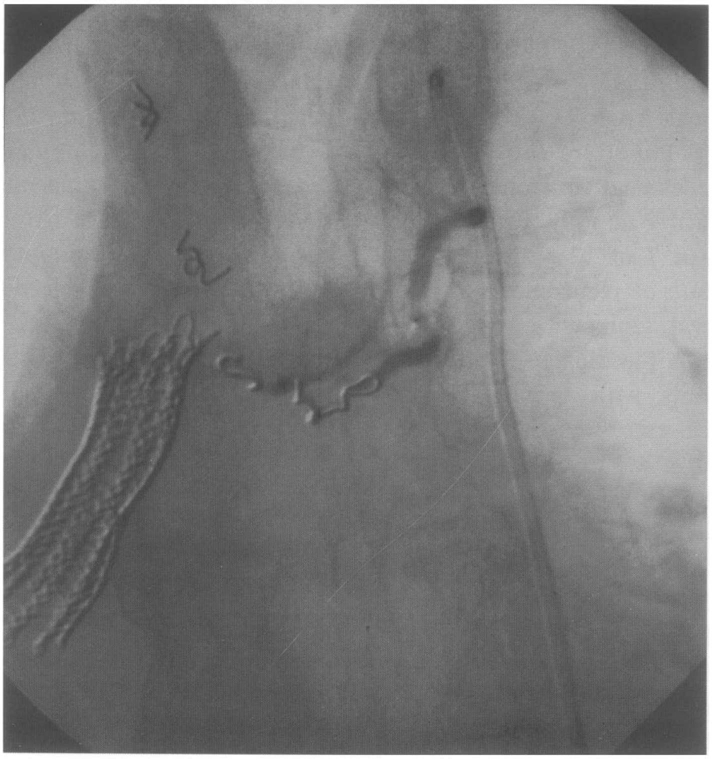

FIGURE 6 Bronchial arteriogram shows vessel occlusion after successful BAE with metal coils (additional finding: Strecker-Stent in the right main bronchus).

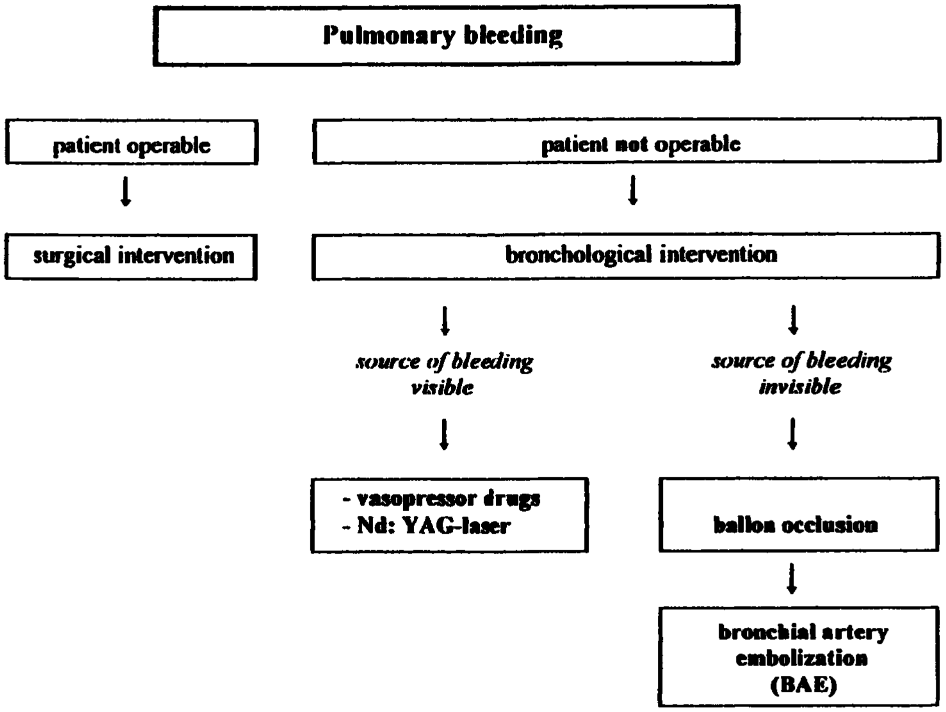

FIGURE 7 Therapeutic procedure in case of pulmonary bleeding.

observation showed reccurence of bleeding in 10 to 44% (Cremaschi *et al.*, 1993).

In patients suffering from bronchial carcinoma, one of the most important causes of pulmonary hemorrhage, clinical outcome is less encouraging. Hayakawa *et al.* (1992) successfully arrested the bleeding in 7 of 12 patients (58%) by means of BAE. Rate of recurrence was 14% in a five-months interval. Our own investigations show interruption of the bleeding in 89% (17 of 19 patients) of patients with bronchial carcinoma, but recurrence rate was 32% in one month follow-up (Witt *et al.*, 1995). These results can be explained in light of the state of the underlying malignancy. Tumor-specific therapy can support the results of BAE and help to reduce bleeding recurrence. Some patients can be transferred to surgery after embolization (Uflacker *et al.*, 1985). In case of recurrence, bronchial arteriography has to be performed to detect recanalization of embolized vessels, development of collaterals, and neovascularisations in active tumors. In every case re-embolization can be performed (Katoh *et al.*, 1990).

Bleeding from pulmonary arteries is usually caused by arteriovenous malformations. Indications for therapy are fistulae increasing in size accompanied by hemoptysis, dyspnoea, and cyanosis, or elevated bleeding risk (Dines *et al.*, 1974; Higgins and Wexler, 1976). Depending on the findings (size, risk factors, operability) pneumological (embolization of pulmonary artery) or surgical (lung resection) interventions are indicated. The high mortality associated with lung resection (4–5%) (Gomes *et al.*, 1989) dictates that embolizaton should be the method of choice, at least in the case of multiple arteriovenous fistulae. In these cases detachable balloons (Figs. 8a and 8b) or micro-metal coils with or without dacron fibres have been employed successfully (Solomon *et al.*, 1991; White *et al.*, 1979).

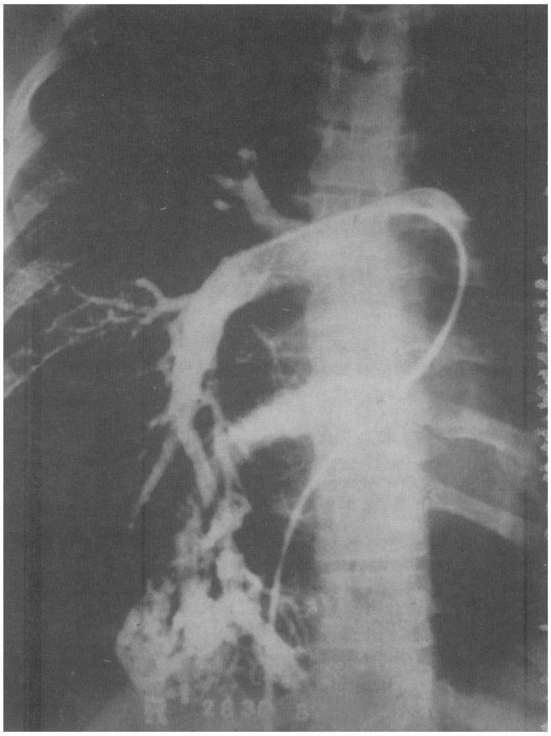

FIGURE 8a Extended arteriovenous fistula in pulmonary angiogram in projection on the right lower lobe.

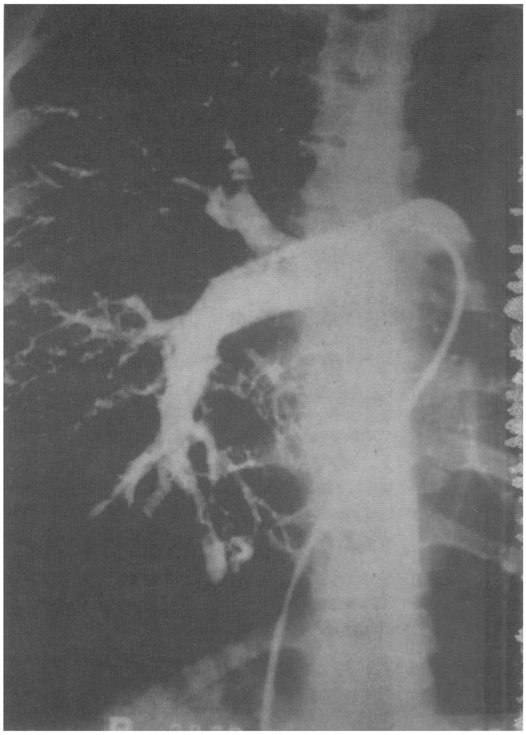

FIGURE 8b Pulmonary arteriogram after successful PTE with detachable balloon.

### *References*

[1] Johnston, H. and Reisz, G. Changing spectrum of hemoptysis. *Arch. Intern. Med.* 1989; **149**: 1666–1668.

[2] Conlan, A.A., Hurwitz, S.S., Krigel, L. *et al.* Massive hemoptysis. *J. Thoracic Cardiovasc. Surg.* 1983; **85**: 120–124.

[3] Corey, R. and Hla, K.M. Major and massive hemoptysis: reassessment of conservative management. *Am. J. Med. Sci.* 1987; **294**: 301–309.

[4] Cauldwell, E.W., Siecket, R.G., Lininger, R.E. *et al.* The bronchial arteries: an anatomic study of 150 human cadavers. *Surg. Gynec. Obstet.* 1948; **86**: 395–412.

[5] Deffabach, M.E., Charan, N., Lakshminarayan, S. *et al.* The bronchial circulation: small but a vital attribute of the lung. *Am. Rev. Respir. Dis.* 1987; **135**: 463–481.

[6] Uflacker, R., Kaemmerer, A., Picon, P.D. *et al.* Bronchial artery embolization in the management of hemoptysis: technical aspects and long-term results. *Radiology* 1985; **157**: 637–644.

[7] Poe, H.R., Israel, H.R., Marin, M.G. *et al.* Utility in fiberoptic bronchoscopy in patients with hemoptysis and a nonlocalizing chest roentgenogram. *Chest* 1988; **92**: 70–75.

[8] McGuinness, G., Beacher, J.R., Harkin, T.J. *et al.* Hemoptysis: Prospective high-resolutuion CT/bronchoscopic correlation. *Chest* 1994; **105**: 1155–1162.

[9] Naidich, D.P., Funt, S., Ettenger, N.A. *et al.* Hemoptysis: CT-bronchoscopic correlation in 58 cases. *Radiology* 1990; **177**: 357-362.

[10] Jackson, C.V., Savage, P.J. and Quinn, D.L. Role of fiberoptic bronchoscopy in patients with hemoptysis and normal chest roentgenogram. *Chest* 1985; **87**: 142–144.

[11] Lederle, F.A., Nichol, K.L. and Parenti, C.M. Bronchoscopy to evaluate hemoptysis in older man with nonsuspicious chest roentgenograms. *Chest* 1989; **95**: 1043–1047.

[12] O'Neil, K.M. and Lazarus, A.A. Hemoptysis. Indications for bronchoscopy. *Arch. Intern. Med.* 1991; **151**: 171–174.

[13] Knott-Craig, C.J., Oostuizen, J.G., Rossouw, G. *et al.* Management and prognosis of massive hemoptysis. Recent experience with 120 patients. *J. Thoracic Cardiovasc. Surg.* 1993; **105**: 394–397.

[14] Witt, Ch., Geisler, A., Ewert, R. *et al.* Bronchoscopy and percutaneous embolization (PTE) of bronchial arteries in severe tumorous haemoptysis. *Eur. Respir. J.* 1995; **8** (Suppl. 19): 178s.

[15] Müller, N.L. Hemoptysis High resolution CT vs bronchoscopy. *Chest* 1994; **105**: 982–983.

[16] Set, P.A.K., Flower, C.D.R, Smith, I.E. *et al.* Hemoptysis: comparative study of the role of CT and fiberoptic bronchoscopy. *Radiology* 1993; **189**: 677–680.

[17] Rabkin, J.E., Vsevolod, I.A., Gothman, L.N. *et al.* Transcatheter embolization in the management of pulmonary hemorrhage. *Radiology* 1987; **163**: 361–65.

[18] Stoll, J.F. and Bettmann, M.A. Bronchial artery embolisation to control hemoptysis: a review. *Cardiovasc. Intervent. Radiol.* 1988; **11**: 263–69.

[19] Newton, Th.-H. and Preger, L. Selective bronchial arteriography. *Radiology* 1965; **84**: 1043–1051.
[20] Wholey, M.H., Chamorro, H.A., Rao, G. *et al.* Bronchial artery embolization for massive hemoptysis. *JAMA* 1976; **236**: 2501–2504.
[21] Vujic, I., Pyle, R., Parker, E. *et al.* Control of massive hemoptysis by embolization of intercostal arteries. *Radiology* 1980; **137**: 617–620.
[22] DiChiro, G. Unintentional spinal cord arteriography. A warning. *Radiology* 1974; **112**: 231–233.
[23] Lamarque, J.L. and Senac, P.J. Die therapeutische Angiographie bei Hämoptysen. *Radiologe* 1979; **19**: 514–520.
[24] Remy, J., Arnaud, S., Fardou, H. *et al.* Treatment of hemoptysis by embolization of bronchial arteries. *Radiology* 1977; **122**: 33–37.
[25] Cremaschi, P., Nascimbene, C., Vitulo, P. *et al.* Therapeutic embolization of bronchial artery: a successful treatment of 209 cases of relapses hemoptysis. *Angiology* 1993; **44**: 295–299.
[26] Hayakawa, K., Tanaka, F., Torizuka, T. *et al.* Bronchial artery embolization for hemoptysis: immediate and long-term results. *Cardiovasc. Intervent. Radiol.* 1992; **15**: 154–159.
[27] Katoh, O., Kishikawa, T., Yamada, H. *et al.* Recurrent bleeding after arterial embolization in patients with hemoptysis. *Chest* 1990; **97**: 541–546.
[28] Dines, D.E., Arms, R.A., Bernatz, P.E. *et al.* Pulmonary arteriovenous fistulas. *Mayo. Clin. Proc.* 1974; **49**: 460–465.
[29] Higgins, C.B. and Wexler, L. Clinical and angiographic features of pulmonary arteriovenous fistulas in children. *Radiology* 1976; **112**: 171–175.
[30] Gomes, M.R., Bernatz, P.E. and Dines, D.E. Pulmonary arteriovenous fistulas. *Ann. Thorac. Surg.* 1989; **7**: 589–592.
[31] Solomon, N., Holey, M.H. and Jarmolowski, C.R. Intravascular stents in the management of superior vena cava syndrome. *Catheter Cardiovasc. Diagn.* 1991; **23**: 245–252.
[32] White, R.I., Kaufman, S.L., Barth, K. *et al.* Embolotherapy with detachtable silicone balloons. *Radiology* 1979; **137**: 619–623.

